# Spatially Explicit Modeling Reveals Cephalopod Distributions Match Contrasting Trophic Pathways in the Western Mediterranean Sea

**DOI:** 10.1371/journal.pone.0133439

**Published:** 2015-07-22

**Authors:** Patricia Puerta, Mary E. Hunsicker, Antoni Quetglas, Diego Álvarez-Berastegui, Antonio Esteban, María González, Manuel Hidalgo

**Affiliations:** 1 Instituto Español de Oceanografía, Centro Oceanográfico de Baleares, Palma de Mallorca, Islas Baleares, Spain; 2 National Center for Ecological Analysis and Synthesis, University of California Santa Barbara, Santa Barbara, California, United States of America; 3 Balearic Islands Coastal Observing and Forecasting System, Palma de Mallorca, Islas Baleares, Spain; 4 Instituto Español de Oceanografía, Centro Oceanográfico de Murcia, San Pedro del Pinatar, Murcia, Spain; 5 Instituto Español de Oceanografía, Centro Oceanográfico de Málaga, Fuengirola, Málaga, Spain; Technical University of Denmark, DENMARK

## Abstract

Populations of the same species can experience different responses to the environment throughout their distributional range as a result of spatial and temporal heterogeneity in habitat conditions. This highlights the importance of understanding the processes governing species distribution at local scales. However, research on species distribution often averages environmental covariates across large geographic areas, missing variability in population-environment interactions within geographically distinct regions. We used spatially explicit models to identify interactions between species and environmental, including chlorophyll a (Chla) and sea surface temperature (SST), and trophic (prey density) conditions, along with processes governing the distribution of two cephalopods with contrasting life-histories (octopus and squid) across the western Mediterranean Sea. This approach is relevant for cephalopods, since their population dynamics are especially sensitive to variations in habitat conditions and rarely stable in abundance and location. The regional distributions of the two cephalopod species matched two different trophic pathways present in the western Mediterranean Sea, associated with the Gulf of Lion upwelling and the Ebro river discharges respectively. The effects of the studied environmental and trophic conditions were spatially variant in both species, with usually stronger effects along their distributional boundaries. We identify areas where prey availability limited the abundance of cephalopod populations as well as contrasting effects of temperature in the warmest regions. Despite distributional patterns matching productive areas, a general negative effect of Chla on cephalopod densities suggests that competition pressure is common in the study area. Additionally, results highlight the importance of trophic interactions, beyond other common environmental factors, in shaping the distribution of cephalopod populations. Our study presents a valuable approach for understanding the spatially variant ecology of cephalopod populations, which is important for fisheries and ecosystem management.

## Introduction

Interaction between species distribution and the environment is a central topic in ecology. Not all species or populations are able to inhabit areas with their most favourable conditions due to the presence of competitors, limited resource availability, anthropogenic impacts and other drivers that may prevent their establishment in certain areas. Therefore, populations of the same species can experience different responses to the environment throughout their distributional range, as a result of spatial and temporal heterogeneity in habitat conditions.

In species distribution models, environmental covariates (e.g. sea surface temperature, SST, in marine species) are often averaged across large geographic areas. By applying average conditions homogeneously over space, important local effects on populations and nonlinear species–environment interactions may not be detected [1,2], and important ecological mechanisms that regulate population abundance and distribution at small scales might remain unknown. However, a deeper understanding of local scale processes governing species distribution and their habitat selection is important for identifying essential areas for survival, reproduction or feeding [3].

Spatially variant or spatially explicit models have become of particular interest in the recent years [3–6], because they can improve our understanding of the interactions between population distributions and environmental influences within geographically distinct habitats or areas. For instance, variable coefficient Generalized Additive Models (vc GAM) have been successfully applied to describe the locally variant effects of temperature on the distribution of several groundfishes [2,7,8] and albacore tuna [6] in the northeast Pacific Ocean.

Despite the utility of spatially explicit models for studying heterogeneity in population abundance, especially in species with highly variable dynamics, they have been applied very rarely to marine taxa other than fish [9,10]. Yet, this modelling approach can be useful for studying cephalopod populations, which are highly sensitive to environmental conditions owing to their short life-cycles and reduced demographic buffering [11]. Like other short-lived species with intrinsically unsteady dynamics, cephalopod populations are rarely regular in abundance and location, displaying different adaptations to local environmental conditions throughout their geographic distribution. For instance, there is high variation in the abundance, biological parameters and life-cycle of *Loligo vulgaris* across the Atlantic Ocean and the Mediterranean Sea, which are attributed to adaptation of populations to large-scale environmental variability [12]. Similar results were recently found for the cephalopods *Illex coindetii*, *Eledone cirrhosa* and *Octopus vulgaris* in the western Mediterranean Sea. The seasonal cycles and the inter-annual distributions differ with geographical location in response to contrasting regional environmental drivers, irrespectively of species-specific life history traits [13,14]. The different adaptations of the cephalopod populations in the Mediterranean Sea are related to the high complexity of this system, which presents diverse hydrodynamic areas [15,16] and different productivity regimes [17,18] at relatively small spatial scales.

In addition to the environmental influence, trophic relationships are one of the main mechanisms that locally affect spatio-temporal abundance and distribution of marine populations. For instance, prey-predator spatio-temporal overlap and prey availability are essential for the survival of predators, as proposed by the match-mismatch hypothesis [19,20]. In turn, these trophic interactions can also be directly or indirectly modified by the environmental forcing. Trophic relationships have been suggested to be no less important than the environment in shaping cephalopod populations [21]. However, the inclusion of predator–prey interactions in cephalopod population models is still rare.

Because recent studies show that temporal variability in the cephalopod populations presents different drivers and responses in neighbouring geographic regions, we hypothesize that spatial variability in the distributions of these species may be sensitive to local variation of drivers influencing the distribution. In this case, the cephalopod populations may present spatially variant effects associated with the variability in environmental and trophic conditions at local scales. Here we aim to develop a spatially explicit modelling approach to (1) identify the spatial variability in the regional distribution of two of the most abundant cephalopods in the western Mediterranean Sea, the squid *Illex coindetii* and the octopus *Eledone cirrhosa* (henceforth referred to as squid and octopus, respectively). (2) In addition to environmental explanatory variables, we test the importance of trophic conditions, i.e. prey densities, as a driver of the variability in spatial distribution patterns. (3) The approach specially focuses in detecting areas where the populations are more sensitive to a given explanatory variable within their distributional range, i.e. spatial local effects.

## Materials and Methods

### Ethic statement

Biological data were obtained from the annual trawl surveys carried out as part of the Mediterranean International Trawl Survey (MEDITS) project. The sampling was performed under repeated international standardized protocol (details of the survey methods can be found in [22]). The surveys were mainly conducted across the Spanish territorial waters in the Mediterranean Sea. The research vessel had full permission from national (Fisheries General Secretariat) and international authorities (General Fisheries Commission for the Mediterranean) to sample in territorial and Mediterranean community waters. No approval by an ethics committee was required as common exploited species were targeted and trawling did not affect endangered or protected species or marine protected areas. Most of the authors participate consistently in the surveys of the MEDITS programme.

### Biological data

**Fig 1:**
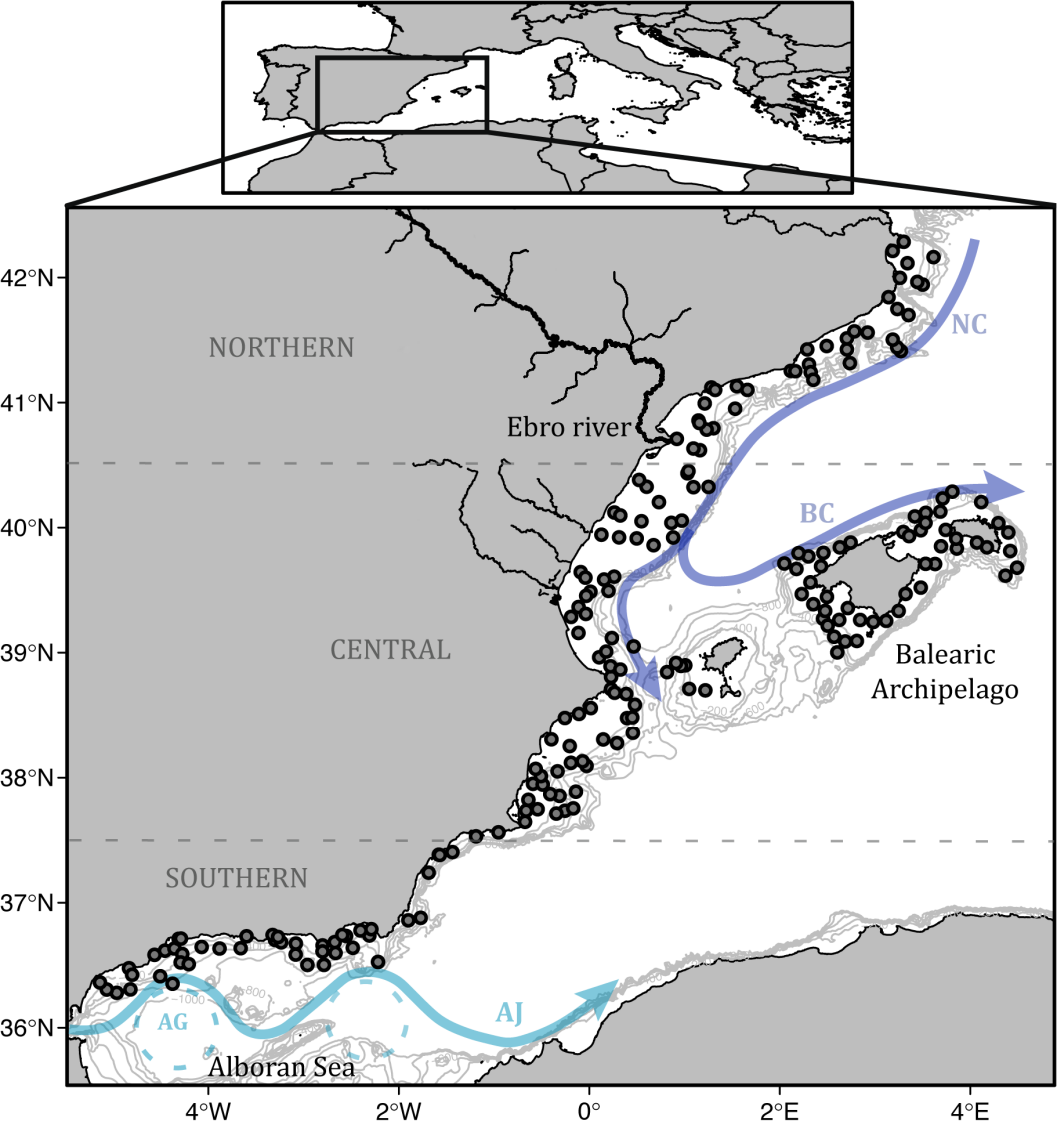
Study area and sampling sites. Map of the western Mediterranean Sea showing 200 to 1000 m isobaths and selected stations sampled for at least 5 out of the 12 years in the MEDITS surveys. Main surface circulation patterns are described by arrows: Northern Current (NC), Balearic Current (BC), Atlantic jet (AJ) and Alboran gyres (AG).

**Fig 2:**
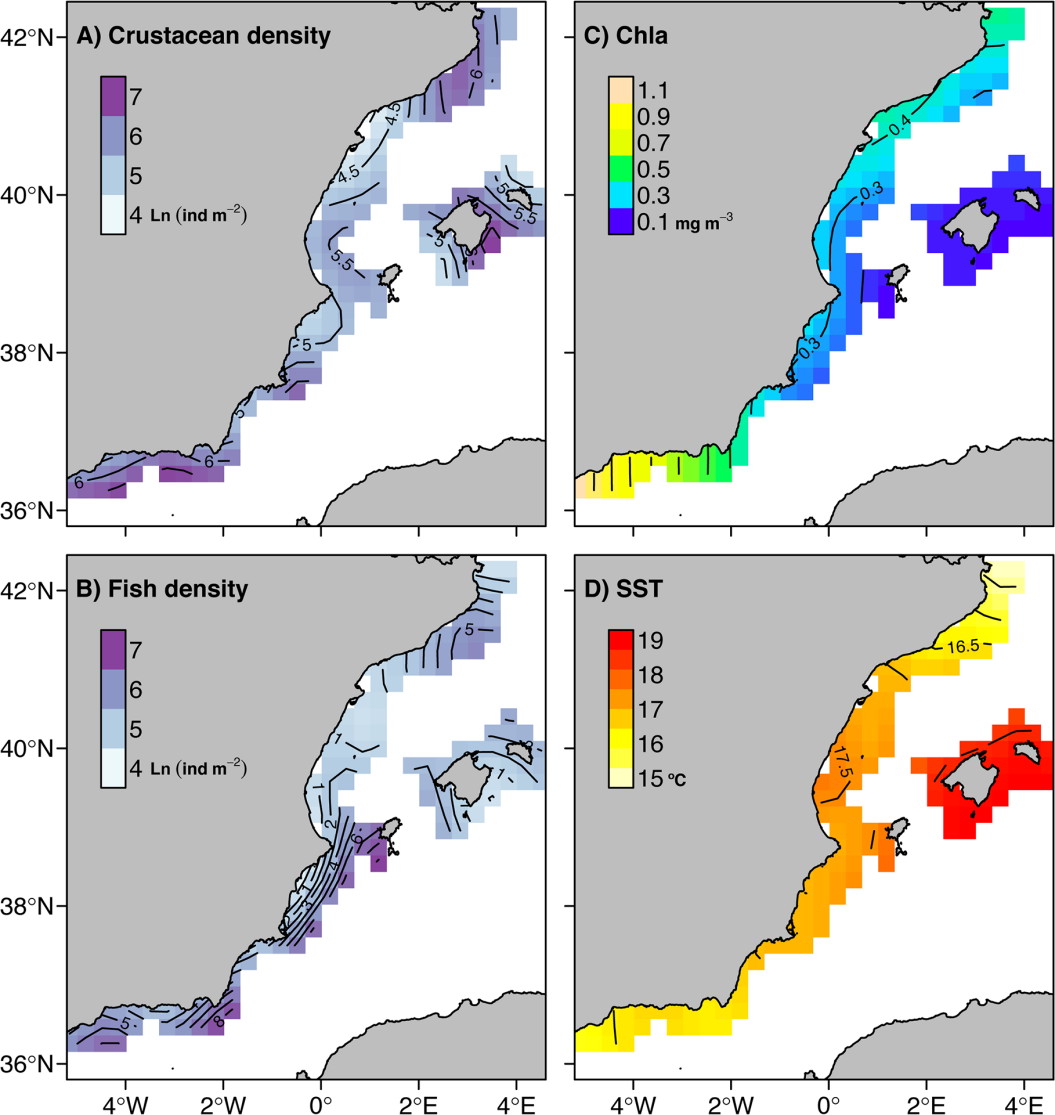
Overall predicted distributions (2001–2012) of environmental and trophic covariates. A) log transformed density of benthic crustaceans (preys of octopus), B) log transformed density of meso-pelagic fish (preys of squid), C) Chlorophyll a concentration (Chla), and D) sea surface temperature (SST) estimated from variable coefficient Generalized Additive Models.

The MEDITS surveys took place between May and July in years 2001 to 2012 in the Spanish western Mediterranean Sea. A similar number of stations were sampled each year (150 annual hauls on average), predefined based on different bathymetric strata (10–50 m, 50–100 m, 100–200 m, 200–500 m and 500–800 m) with approximately replicated locations (Fig 1). An experimental trawl net (GOC 73) was designed for the scientific purposes of the surveys. The gear was tested for the catchability of common benthic and pelagic species at the beginning of MEDITS programme that lead to implement some technical improvements (Bertrand et al 2002). The cod-end is 20 mm mesh size, with ca. 17 and 2.8 m of horizontal and vertical openings, respectively. These characteristics ensure higher catchability of demersal species than those obtained by gears use in commercial fisheries. Sampling information (date, time, position, depth, duration, distance trawled, vertical and wing opening of the net) and species biological data (weight, number, sex and length) were routinely recorded. We restricted our analysis to those stations that were sampled during at least 5 out of the 12 years. Species abundances were standardized using sampling information to obtain density values in individuals per km^2^. Cephalopod prey densities were also estimated (in individuals per km^2^) as a potential driver influencing the spatial distributions of the two cephalopod species. Based on stomach content analyses (see methodology in [23]) of 134 octopus and 265 squid (S1 Table), we identified broad prey groupings for the two species. In addition, species of similar size, depth and habitat distribution as those identified in stomach contents and those encompassed in high taxonomic levels of identification (e.g. species in family Paguridae) were included as potential prey items (S2 Table). In general terms, the diet of octopus consists of benthic crustaceans, mainly crabs, while squid mostly prey on myctophids and other small meso-pelagic fishes. Total densities of cephalopod prey were calculated as the summed densities of all potential prey species occurring at each sampling location (Fig 2A and 2B).

### Environmental data

Other putative environmental drivers, i.e. surface Chla (mg m^-3^) and SST (°C) derived from satellite remote sensing, were also included in our analysis. Remotely sensed data were obtained from NOAA’s CoastWatch Program (available: http://coastwatch.noaa.gov/. Accessed 03 June 2014) and NASA's Goddard Space Flight Center (available: http://www.nasa.gov/centers/goddard/. Accessed 05 June 2014) using different sensors to match the time range of the sampled data. Chla datasets were obtained from Sea WiFS (2001 to 2002) and MODIS sensors (available since 2003) and processed with standard color algorithms [24]. Whereas AVHRR (2001–2002) [25,26] and MODIS (2003–2012) [27] supplied SST datasets. Differences in measurements between sensors are relatively minor [28,29], displaying no important impact on the results. Values of Chla and SST in a 9 km radius around each sampled location were extracted from 8-day composites and 4 km resolution files. From these values, monthly averages (previous to the date of sampling) of Chla and SST were calculated at each sampled location (Fig 2C and 2D). The chosen spatial and temporal resolution is appropriate for identifying the local productivity regimes and surface oceanographic processes in the study area, while minimizing cloud impact on the measurements.

### Modelling approach

Due to the high proportion of zeros in the observations of both species (~50%), we used Delta Generalized Additive Models (GAM) [30,31] to estimate cephalopod distributions. The Delta-GAM includes two different sub-models [32,33]. First, presence-absence data are modelled using a binomial logit GAM (stage 1, *P*_*ij*_) and second, only positive density values are used in a Gaussian GAM with a log link function (stage 2, *D*_*ij*_). Finally, to reduce bias of the sub-models and obtain the overall predictions of Delta-GAM (*Y*_*ij*_) the results of both model fits are multiplied (*Y*_*ij*_ = *P*_*ij*_ * *D*_*ij*_ *)*. For model selection (see below), the same set of covariates was initially included in stages 1 and 2 of the Delta-GAM for simplicity.

Starting from the Delta-GAM, we developed and contrasted two different model formulations: a full Delta-GAM and a variable coefficient Delta-GAM (vc Delta-GAM). The full Delta-GAM assumes that changes in distribution are homogeneous over space and time, and the effects of covariates are independent and additive. Variable coefficient Delta-GAM, by contrast, tests the spatially variant effects of environmental and trophic drivers on cephalopod distributions. This last formulation assumes that the relationship between response (occurrence or density of a species) and covariates is locally linear, but the coefficients of regression are allowed to change smoothly in relation to the geographical position. The formulation of the two approaches (including stages 1 and 2 in both Delta-GAM) is as follows:

A) A full Delta-GAM of the form:

$$C_{y(\phi ,\lambda )}=a_{y}+s_{1}\left(\phi ,\lambda \right)+s_{2}\left(depth\right)+s_{3}{\left(DOY\right)}_{y(\phi ,\lambda )}+s_{4}{\left(prey\right)}_{y(\phi ,\lambda )}+s_{5}{\left(Chla\right)}_{y(\phi ,\lambda )}+s_{6}{\left(SST\right)}_{y(\phi ,\lambda )}$$

where *C* is either the probability of cephalopods occurrence (stage 1, *P*_*ij*_) or an estimate of natural logarithm of density when the cephalopods are present (stage 2, *D*_*ij*_). Smoothing functions are denoted by *s*; *a*_*y*_ is the year (*y*)-specific intercept, geographical position was described by latitude (*ϕ*) and longitude (*λ*), *DOY* indicates the day of the year, *prey* denotes the natural logarithm of prey densities and *Chla* and *SST* are expressed in monthly mean values.

B) A variable coefficient Delta-GAM of the form:

$$\begin{array}{l} C_{y(\phi ,\lambda )}=a_{y}+s_{1}\left(\phi ,\lambda \right)+s_{2}\left(depth\right)+s_{3}{\left(DOY\right)}_{y(\phi ,\lambda )}+s_{4}\left(\phi ,\lambda \right)prey_{y(\phi ,\lambda )}\\ +s_{5}\left(\phi ,\lambda \right)Chla_{y(\phi ,\lambda )}+s_{6}\left(\phi ,\lambda \right)SST_{y(\phi ,\lambda )} \end{array}$$

in which environmental and trophic covariates (Chla, SST and preys) are tested for potential spatially explicit effects on cephalopod occurrence and density, while the rest of the covariates remained as common smooth terms. To describe the potential spatially explicit effects on cephalopod densities, local coefficients of regression (slopes) of these terms were extracted from the sub-model performed on positive abundance data (stage 2). The values of significant slopes (based on 95% confidence interval) reflect the strength of the effect of a given covariate in cephalopod densities at each geographical position.

All possible combinations of the covariates described above were tested in the model selection. This selection process was applied independently for each stage (occurrence and density) and model formulation (full Delta-GAM and vc Delta-GAM). Independent model structures were used since drivers affecting the presence of a given species are not necessarily the same that influence its abundance [32,34–36]. An independent model selection approach for presence-absence and abundance information is able to address those different drivers and provide better fit of each type of data set. To compare full and reduced versions of the models, we used the Akaike Information Criterion (AIC) as a measurement of goodness of fit, and the genuine Cross Validation (gCV) [37] as a measure of the out-of-sample predicted mean squared error. The lowest values of both criterions determined the model that best explained the variance of the response and was optimal for predictions. All analyses were conducted using *mgcv* and *MuMIn* packages in R software, version 3.1.1 [38].

## Results

### Spatial modelling approach

For both cephalopods, the vc Delta-GAM performed better than the full Delta-GAM formulations in terms of AIC, gCV and deviance explained (Table 1). Differences in model fit between the two formulations were relatively minor for octopus, but vc Delta-GAM notably improved the model fit for squid. The best fit in presence-absence vc GAM for octopus explained 37.4% of the deviance, which included, in addition to the base parameters of the models (year, position and depth), the density of prey items and Chla concentration as significant predictors (Table 1). Similarly, day of the year, density of prey items, Chla and SST were included in the presence-absence best sub-model for squid, which explained 33.1% of deviance in its occurrence (Table 1). The density sub-model of the vc GAM explained 32.5% and 43% of the deviance for octopus and squid, respectively. The three spatially variant terms, (prey density, Chla and SST) were retained in density sub-models that best fit the density distributions of the two species (Table 1).

**Table 1:** Model selection for the stages (sub-models) of Delta Generalized Additive Models in octopus (*Eledone cirrhosa)* and squid (*Illex coindetii)*. Comparisons of full Delta-GAM and variable-coefficient, vc, Delta-GAM (formulations were simplified). Base term includes *C*_*y*,(*ϕ*,*λ*)_ = *a*_*y*_ + *s*_1_(*ϕ*,*λ*) + *s*_2_(*depth*); where *C*_*y*,(*ϕ*,*λ*)_ is either the probability of cephalopods occurrence (stage 1, presence-absence sub-model) or an estimate of natural logarithm of density when the cephalopods are present (stage 2, density sub-model); *a* is the year (*y*) -specific intercept, latitude *ϕ*, longitude *λ* and depth. Other parameters included as smoothing functions (*s*_*1-6*_) in the models are prey, as the natural logarithm of prey densities, day of the year, DOY, monthly average chlorophyll a concentration, Chla and sea surface temperature, SST. For each model and stage AIC: Akaike Information Criterion, AIC: increment of AIC: ΔAIC, gCV: genuine Cross Validation and dev. exp: percentage of deviance explained by the model are given. Best models obtained for each formulation and stages are in bold.

Species	DeltaGAM	Stage	Model terms	AIC	ΔAIC	gCV	Dev.exp
*E*. *cirrhosa*	full	1	Base + s_3_(DOY) + s_6_(SST)	1595.27	0	0.16	37.1
	full	1	**Base + s**_**3**_ **(DOY)**	**1595.27**	**0**	**0.16**	**36.9**
	full	1	Base + s_6_(SST)	1595.88	0.61	0.16	37
	full	2	Base + s_4_(prey) + s_5_(Chla) + s_6_(SST)	2486.96	0	0.81	32.2
	full	2	**Base + s**_**4**_ **(prey) + s**_**5**_ **(Chla)**	**2488.67**	**1.70**	**0.80**	**31.6**
	full	2	Base + s_3_(DOY) + s_4_(prey) + s_5_(Chla) + s_6_(SST)	2488.89	1.93	0.81	32.2
	vc	1	Base + s_3_(DOY) + s_4_(ϕ, λ)·prey + s_5_(ϕ, λ)·Chla	1590.46	0	0.16	37.5
	vc	1	**Base + s**_**4**_ **(ϕ, λ)·prey + s**_**5**_ **(ϕ, λ)·Chla**	**1590.83**	**0.37**	**0.16**	**37.4**
	vc	1	Base + s_3_(DOY) + s_4_(ϕ, λ)·prey	1591.81	1.35	0.16	37.2
	vc	2	Base + s_3_(DOY) + s_4_(ϕ, λ)·prey + s_5_(ϕ, λ)·Chla + s_6_(ϕ, λ)·SST	2482.53	0	0.82	32.8
	vc	2	**Base + s**_**4**_ **(ϕ, λ)·prey + s**_**5**_ **(ϕ, λ)·Chla + s**_**6**_ **(ϕ, λ)·SST**	**2483.45**	**0.92**	**0.81**	**32.5**
	vc	2	Base + s_3_(DOY) + s_4_(ϕ, λ)·prey + s_5_(ϕ, λ)·Chla	2486.53	4	0.82	32.3
*I*. *coindetii*	full	1	**Base + s**_**3**_ **(DOY) + s**_**5**_ **(Chla) + s**_**6**_ **(SST)**	**1756.49**	**0**	**0.18**	**31.2**
	full	1	Base + s_3_(DOY) + s_4_(prey) + s_5_(Chla) + s_6_(SST)	1757.02	0.53	0.18	31.3
	full	1	Base + s_3_(DOY) + s_6_(SST)	1758.85	2.35	0.18	31
	full	2	Base + s_3_(DOY) + s_4_(prey) + s_5_(Chla) + s_6_(SST)	2774.77	0	1.4	38.5
	full	2	**Base + s**_**4**_ **(prey) + s**_**5**_ **(Chla) + s**_**6**_ **(SST)**	**2775.84**	**1.07**	**1.4**	**38.2**
	full	2	Base + s_3_(DOY) + s_4_(prey) + s_5_(Chla)	2778.64	3.87	1.4	37.8
	vc	1	**Base + s**_**3**_ **(DOY) + s**_**4**_ **(ϕ, λ)·prey + s**_**5**_ **(ϕ, λ)·Chla + s**_**6**_ **(ϕ, λ)·SST**	**1720.97**	**0**	**0.18**	**33.1**
	vc	1	Base + s_3_(DOY) + s_4_(ϕ, λ)·prey + s_6_(ϕ, λ)·SST	1723.52	2.55	0.17	32.7
	vc	1	Base + s_4_(ϕ, λ)·prey + s_5_(ϕ, λ)·Chla	1730.69	9.72	0.18	32.3
	vc	2	**Base + s**_**4**_ **(ϕ, λ)·prey + s**_**5**_ **(ϕ, λ)·Chla + s**_**6**_ **(ϕ, λ)·SST**	**2712.25**	**0**	**1.30**	**43**
	vc	2	Base + s_3_(DOY) + s_4_(ϕ, λ)·prey + s_5_(ϕ, λ)·Chla	2713.26	1.01	1.32	42.6
	vc	2	Base + s_3_(DOY) + s_4_(ϕ, λ)·prey + s_5_(ϕ, λ)·Chla + s_6_(ϕ, λ)·SST	2713.96	1.71	1.32	43

### Spatial distribution of cephalopod species

**Fig 3:**
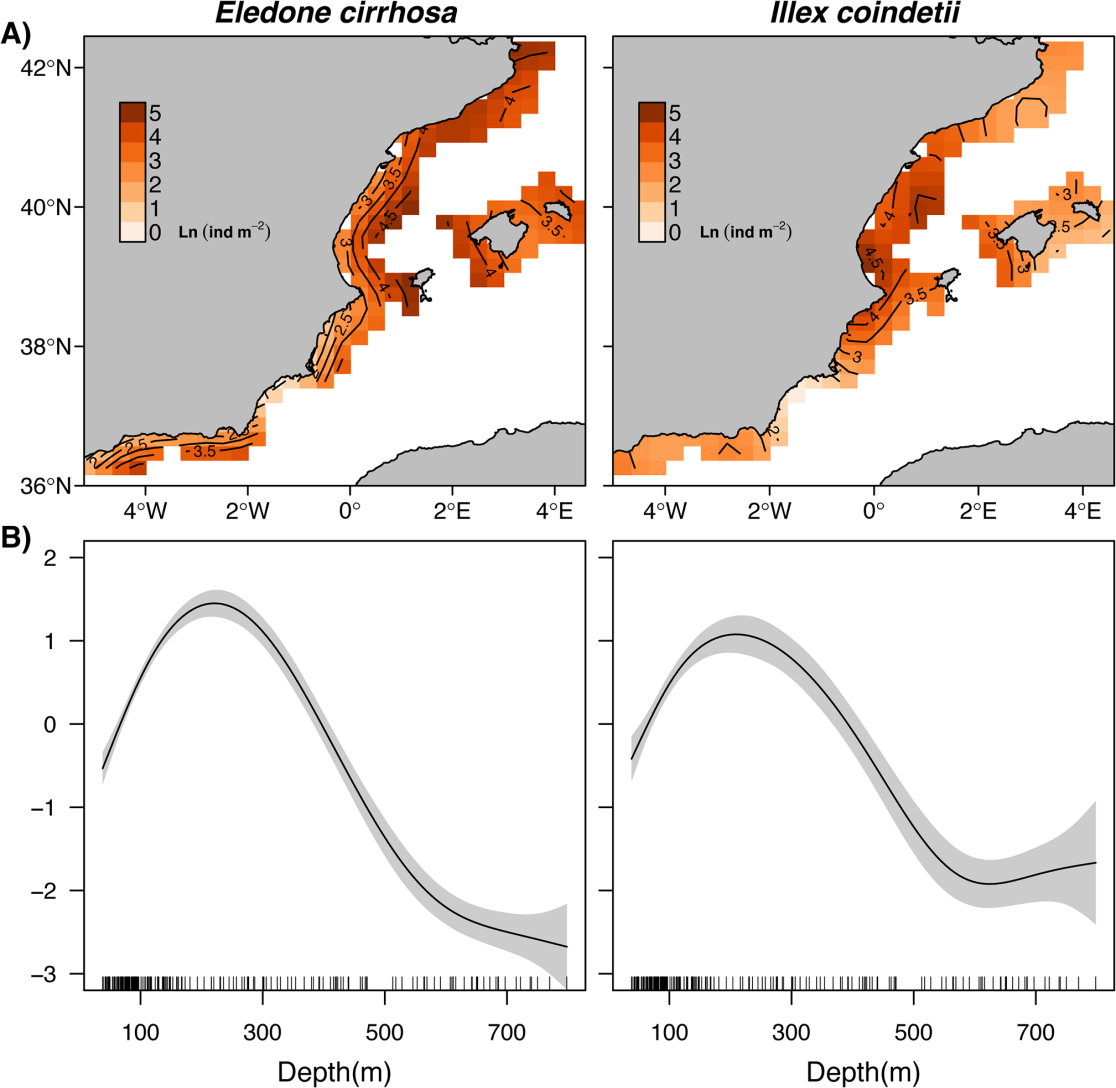
Overall predicted distributions of octopus (*Eledone cirrhosa*) and squid (*Illex coindetii*) estimated from variable coefficient Delta Generalized Additive Models. A) Spatial distribution of log transformed densities. B) Partial effects of depth on the vertical distribution of cephalopod densities. Shaded areas indicate 95% confidence intervals.

The distributions predicted by the best models confirmed different spatial patterns between octopus and squid (Fig 3). We found high probabilities of occurrence of both species across most of the study area. Low occurrences of both cephalopods were only found in the southern region (Alboran Sea). In contrast, the areas with the highest densities (considering both stages of the best vc Delta-GAM) differed considerably between the two species. The highest densities of octopus were located in the northern region, continuing throughout the offshore waters of southern Ebro river delta and the west shelf of the Balearic Archipelago (Fig 3A). In the case of squid, areas with the highest densities were more limited to the central region, southwards Ebro river delta (Fig 3A). Density values of squid decreased with distance from the central region and high-density areas exhibited less variability in environmental conditions than those occupied by octopus. For both species, the lowest densities were predicted in the southern region, which is mainly characterized by very high Chla concentrations (Fig 2C). Depth had a positive effect on both the occurrence and densities of the two cephalopods. Maximum densities were found between 200–250 m depth, while negative effects were observed at depths lower than 50 m and higher than 400 m (Fig 3B).

### Spatially explicit effects of environmental and trophic covariates

**Fig 4:**
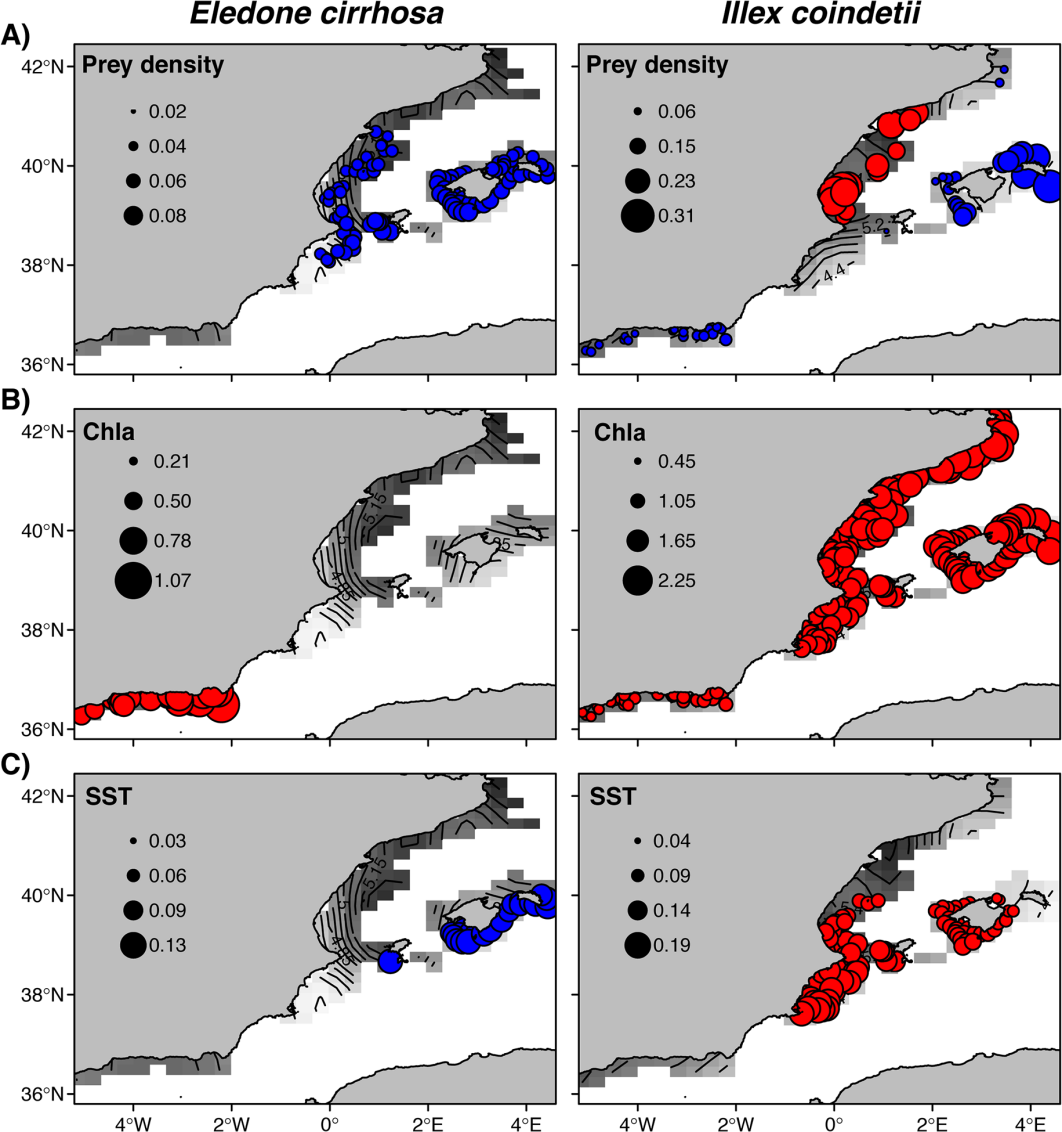
Spatially explicit effects of environmental and trophic covariates on the density of octopus (*Eledone cirrhosa*) and squid (*Illex coindetii*). Effects of A) prey densities, B) chlorophyll a concentration (Chla) and C) sea surface temperature (SST) estimated from variable coefficient Generalized Additive Models using only positive data. Red and blue bubbles represent respectively negative and positive effect of each covariate on log-transformed cephalopod densities. Only effects (regression slopes) significantly different from zero are showed, based on the estimates of the 95% confidence interval. Overall predicted densities (log transformed) of each species are shown underlayed, with the highest densities indicated by dark grey cells.

We observed significant spatially explicit effects of prey density, Chla and SST on the local density of both cephalopod species (Fig 4). For octopus, the slopes for prey density showed a weak but positive effect in the central region and around the islands (Fig 4A), where intermediate to low prey densities were found (Fig 2A). A notable negative effect of Chla on population density was observed in the southern region (Fig 4B), where the highest values of Chla were recorded (Fig 2C). In contrast, SST showed a localized positive effect on octopus densities only observed in the southern locations of the Balearic Archipelago (Fig 4C).

Very different spatially explicit effects were observed for squid. A positive influence of prey densities on squid was found in the southern region and around the Balearic archipelago (Fig 4A). These effects were much higher in the islands, especially in the western part. Additionally, important negative effects of prey density were detected in the central region surrounding the Ebro river delta. Chla revealed a general and high negative effect for squid densities in the whole study area (Fig 4B). Considerable effects of SST were also found for this species, with local negative slopes in the western part of the archipelago and in the central region, displaying an evident north-south gradient in the strength of the effect (Fig 4C). These regions recorded the highest temperature values of the study area (Fig 2D).

## Discussion

### Spatial modelling approach

The spatially explicit modelling approach developed in the present study allowed us to examine the heterogeneity in the spatial abundances of the squid *Illex coindetii* and the octopus *Eledone cirrhosa* populations in the western Mediterranean Sea. Our results revealed important local species–environment interactions that drive the abundance and distributional patterns in this geographically and oceanographically complex system. The inclusion of delta models in the approach showed that ecological processes governing presence-absence and densities are partially independent for octopus, but this is not necessarily a general pattern, as we observed in squid. The best model obtained for octopus included different covariates in each of the model stages. While the occurrence was determined by the presence of productive areas (prey densities and Chla), densities were also influenced and SST, which usually dictate recruitment peaks in the seasonal cycle of this species [39]. In contrast, prey densities, Chla and SST were included in both stages for squid, with the day of the year only retained in the presence-absence sub-model, suggesting that similar processes determine both the occurrence and abundance of this species.

### Spatial distribution of cephalopod species

One of the most novel results of our study is the contrasting regional distribution pattern obtained for each cephalopod species, observed by combining information from the Balearic Archipelago and the mainland. These contrasting patterns elucidate a species-specific adaptation to the main trophic pathways derived from the bottom-up forcing of primary production regimes observed in the western Mediterranean: the southwards flow of high productive waters originated in the north-western upwelling (the Gulf of Lion) in the case of the octopus and the bottom-up processes triggered by the Ebro river discharges in the case of the squid. The highest occurrence and densities of octopus were located in the northern region and continue towards south-west across the offshore waters and the western shelf of the Balearic Archipelago. This distribution follows the Mediterranean Northern Current pattern (Fig 1), which spreads highly productive waters from the phytoplankton bloom produced by the autumn-winter upwelling from the Gulf of Lions [18]. This makes this area one of the most productive regions in the western Mediterranean Sea, even though it was characterized by low chlorophyll concentrations in spring (as observed in data). A time-lagged response to the upwelling and the dispersion of highly productive waters clearly explains the octopus distribution we found in spring months. This is in accordance with previous research reporting delayed responses of several months to surface environmental conditions (e.g. Chla) in octopus populations from the western Mediterranean [13,40,41].

By contrast, the highest densities of squid were located beneath the Ebro river mouth, in the area influenced by the river plume. Ebro run-off is highest during spring [42,43], but ephemeral discharges depend on wind and rainfall conditions, which can generate intermittent bottom-up production processes. In spring, the solar heating and the decrease in wind activity create a thermocline that inhibits vertical mixing and a depletion of surface nutrients. Therefore, the only source that may contribute to surface primary production, enhancing the trophic cascade, is nutrient inputs from river discharges [43]. Contrary to the benthic octopus, the nektobenthic squid responds to co-occurring conditions due to its stronger association with the water column [13]. Therefore, food availability in the river plume can favour squid recruitment, as have been previously observed in cephalopod populations [40,44,45], including squid [13]. This does not precludes that at local scale, octopus might also be partly influenced by the river discharges [13,40,41].

The southern region (Alboran Sea) presented very low occurrences and densities of both cephalopod species. This region is characterized by a turbulent mixing due to exchanges of Mediterranean and Atlantic waters and persistent anticyclonic gyres (Fig 1) that create highly oligotrophic conditions. However, the incoming Atlantic jet strongly enhances primary and secondary production in surface waters around the gyres [46], resulting in very high Chla levels in comparison to the other study regions. The instability in primary production and hydrographic conditions might make this area less suitable to cephalopod populations. A non-exclusive explanation might be related to differences in seasonal dynamics of cephalopod populations in the western Mediterranean [14]. Populations from the southern region might display a different seasonal cycle to cope with such unsteady conditions, as suggests the fact that the lowest densities of *Octopus vulgaris* in this area were observed during spring [47].

### Spatially explicit effects of environmental and trophic covariates

As we hypothesized, spatially explicit effects associated with the variability of environmental and trophic drivers were found in the two cephalopod species across the study area. Prey densities describe the direct link of potential food availability for adult individuals, while Chla is a proxy of productivity and energy flow in the trophic pathway that ultimately influences the yield of upper trophic levels (indirect link). By contrast, there are multiple mechanistic linkages between SST and population distributions, which effects can be interpreted from the physiological to population perspective [11,48,49]. Since the highest densities of the two cephalopods were found between 100–400 m depth, where temperature remains constant during spring (around 13°C [44]), physiological processes should not influence the observed patterns [35]. Temperature is also directly or indirectly associated with the availability of food resources and consumption rates in marine food-webs [50], as has been observed in most organisms including cephalopods [51–53].

Prey availability usually constitutes the foremost condition for habitat selection or aggregative response of predators [54,55]. Our results showed, however, that this seems to apply to the distribution of octopus but not to that of squid. Food limited areas can be inferred from spatially explicit effects of prey observed in the two cephalopods. Scarce prey availability was observed for octopus in the Balearic Archipelago and the central region of the mainland, as reflected in the small positive effects of prey abundances. This agrees with distributional maps in those areas, where high densities of octopus matched intermediate prey densities. By contrast, food availability strongly limited squid densities around the archipelago, in accordance with the observed low abundances of both prey and predator. The strength of this effect showed a west-east gradient in the archipelago, suggesting a stronger influence towards the edge of the distribution area of the squid. In another sub-optimal distribution area, such as the Alboran Sea, weak positive effects of prey densities were also observed for squid. Despite the high prey availability recorded in this region, very low densities of squid were found, which might reflect unsteady trophic interactions between prey and predator or the influence of other factors in this highly dynamic oceanographic area. Besides positive local effects of prey density, the spatially explicit approach also allowed identifying the opposite situation for squid in the central region of the mainland around the Ebro river plume. As previously mentioned, river discharges make this area very productive locally, especially for the pelagic system [43,56]. We suggest that the local decrease in squid abundance when high prey densities occur are due to the increase of pelagic competitors and predators because of the high primary production in this region [57,58]. This could explain why we found the high-density distribution of squid in this area, in spite of the lowest prey abundances and the negative effects of prey. It is also worth noting that the small meso-pelagic fish preyed on by squid usually display low catchability with the trawling gear, whereby their abundance values might be biased.

A common pattern was observed in the two species related to Chla, which showed negative effects on cephalopod abundances despite their high-density distributions matching areas of high productivity. Although this might seem counterintuitive, it would be in accordance with the argument of competition pressure described above. The food-web in the western Mediterranean is mainly controlled by small pelagic fishes and changes in their biomass have important consequences for all trophic levels [57,58], especially when food becomes limiting as in oligotrophic seas such as our study area [57]. This seems to be the case for squid, as the negative effect of Chla is widespread in the whole study area. Competition pressure with pelagic fish may indirectly affect inter-annual variability of squid, as primary production enhance fish populations that compete more effectively with the early and juvenile stages of squid, thereby inducing a decrease in their regional density. By contrast, densities of octopus were only negatively influenced by Chla in the Alboran Sea. Although Chla presents more influence in the pelagic than in the benthic system, the high hydrodynamics is also associated with instability in trophic interactions [11]. Therefore, unstable trophic interactions, especially in a sub-optimal area of distribution as this one, could also affect the benthic system and the octopus densities.

Several studies demonstrate that species inhabiting the boundaries of their distributional areas display higher sensitivity to environmental variability [59]. In the present study, SST effects were observed in the distribution limits of both species, where the highest temperatures and oligotrophy levels were recorded. However, opposite effects were found in both species related to SST. Squid populations were negatively affected by temperature. Warmer temperatures create stronger and longer stratified waters by limiting the input of nutrients along with phytoplankton and zooplankton growth. Additionally, inter-annual and seasonal variability in zooplankton abundance show a clear response to warmer periods by reducing biomass and changing the composition and structure of communities [60]. That results in important implications on the productivity and the functioning of the pelagic ecosystem in the western Mediterranean [61], which can finally limit or reduce the abundance of squid in the warmest areas. The strength of the effect also showed a north-south gradient, probably related to the spreading of north-western highly productive waters. As described above, the northern current spreads colder and productive waters southwards, and forms a branch throughout the northern slope of the Balearic Archipelago (Fig 1). Therefore, the effects of stratification seem to be lower in the north than in the south of the islands. This is in agreement with the contrasting regimes and species responses [62–64], including cephalopods [13,14], observed between both sides of the archipelago. By contrast, octopus showed positive effects of temperature only in the southern Balearic Archipelago. Warmer and more saline waters coming from the Atlantic (AW) are established in this area, having implicitly associated the lowest primary and secondary production in the western Mediterranean. While it is difficult to elucidate how warm temperature benefits octopus densities, we suggest that the most plausible mechanism might be related to the links between surface conditions and benthic environments observed during early summer in the Balearic Islands. When cold western Winter Intermediate Waters (WIW) are present in the channel between islands due to colder winters, northwards progress of the warmer AW throughout the channels is blocked in spring [65]. WIW are also linked to surface AW dynamics and the formation of a mesoscale front in the south of the islands. Inter-annual variation in the presence or absence of the WIW can also modify the temperatures in surface and intermediate waters, and affect the local productivity, planktonic communities [66,67] and meso-pelagic fish [68] associated with the front. Together with temperature, these mechanisms might directly or indirectly explain the effects observed in octopus densities. However, additional factors not included in our work such as substrate, predation or fishing, cannot be discarded, since they might be also influenced by temperature.

## Conclusions

Spatially variant models lead us to better understand cephalopod distributions in heterogeneous and complex systems, such as the western Mediterranean Sea. The spatial heterogeneity in abundance observed in the octopus *Eledone cirrhosa* and the squid *Illex coindetii* populations was ascribed to different trophic pathways present in the study area. Contrasting spatially explicit effects were observed in the two species, with stronger effects mostly found in the limits of their distributional range. Additionally, results highlight the importance of trophic interactions, in addition to environmental factors, in shaping cephalopod distributions in a highly oligotrophic system such as the Mediterranean Sea. Local adaptations of cephalopod populations to environmental and trophic conditions were evidenced, suggesting that complex population structures and dynamics are more widespread than expected [13,14,21].

Our study highlights that the knowledge of environmental and trophic effects in the abundance and distribution of cephalopod populations at regional and local scales is needed for marine spatial planning, conservation and management. Additionally, the modelling approach used here can be applied in future investigations of biological responses to climate change, which is expected to induce shifts in marine species distributions and abundances including cephalopods [69–71]. This is paramount in the Mediterranean Sea, where marine populations and food-webs are especially vulnerable to climate change [72–74] and highly dependent on favourable environmental and trophic conditions at small spatial scales [14,57,75].

## Supporting Information

S1 Table Diet composition of octopus (*Eledone cirrhosa)* and squid (*Illex coindetii)*. Frequency of occurrence (%F) of species identified to the lowest possible taxon in stomach contents.
(TIF)

S2 Table Main prey species selected for octopus (*Eledone cirrhosa)* and squid (*Illex coindetii)*. The frequency of occurence (%F) and cumulative densities (%C.Den) in relation to all potential prey species selected are shown for the 10 main preys found in the MEDITS surveys from 2001 to 2012.
(TIF)
