# Supplementary figures and images for: Spatially Explicit Modeling Reveals Cephalopod Distributions Match Contrasting Trophic Pathways in the Western Mediterranean Sea
Source: PLoS One. 2015 Jul 22;10(7):e0133439. doi: 10.1371/journal.pone.0133439 (PMC4511516; doi:10.1371/journal.pone.0133439)

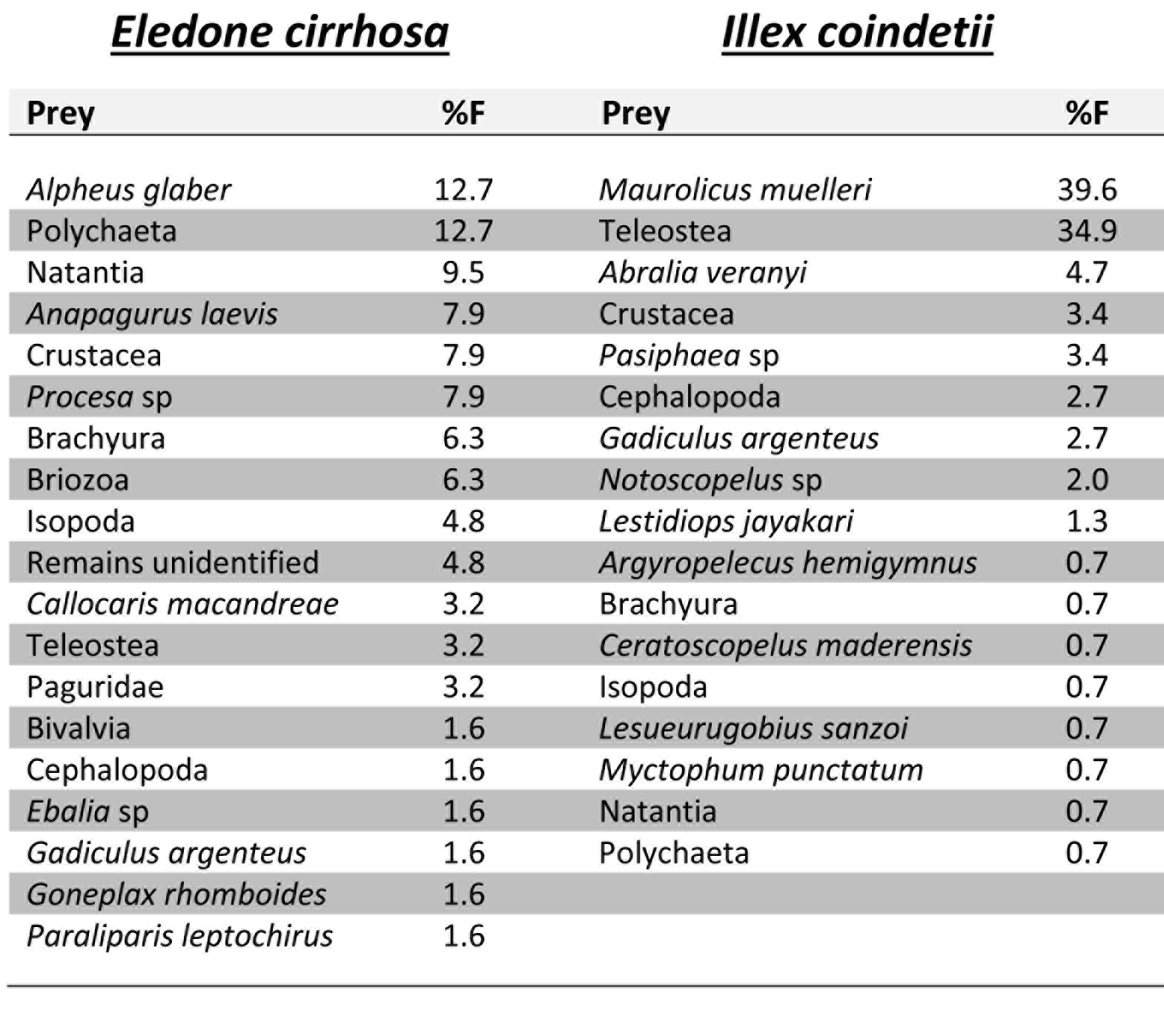

Supplement: S1 Table — Frequency of occurrence (%F) of species identified to the lowest possible taxon in stomach contents. (TIF) [file pone.0133439.s001.tif]

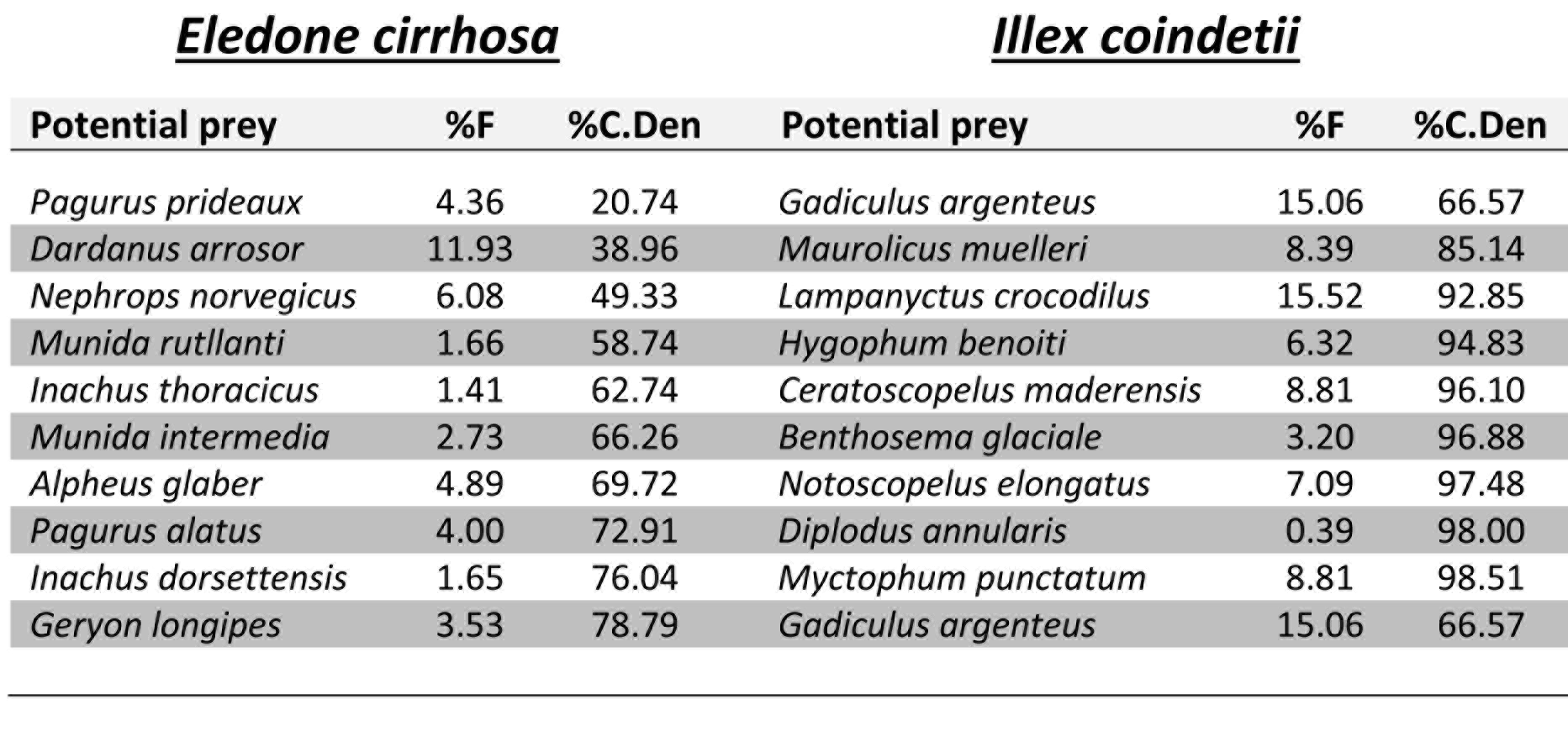

Supplement: S2 Table — The frequency of occurence (%F) and cumulative densities (%C.Den) in relation to all potential prey species selected are shown for the 10 main preys found in the MEDITS surveys from 2001 to 2012. (TIF) [file pone.0133439.s002.tif]
